# Specifications of standards in systems and synthetic biology: status and developments in 2022 and the COMBINE meeting 2022

**DOI:** 10.1515/jib-2023-0004

**Published:** 2023-03-29

**Authors:** Matthias König, Padraig Gleeson, Martin Golebiewski, Thomas E. Gorochowski, Michael Hucka, Sarah M. Keating, Chris J. Myers, David P. Nickerson, Björn Sommer, Dagmar Waltemath, Falk Schreiber

**Affiliations:** Institute for Biology, Institute for Theoretical Biology, Humboldt-University Berlin, Berlin, Germany; University College London, London, UK; Heidelberg Institute for Theoretical Studies (HITS), Heidelberg, Germany; School of Biological Sciences, University of Bristol, Bristol, UK; California Institute of Technology, Pasadena, USA; Department of Electrical, Computer, and Energy Engineering, University of Colorado Boulder, Boulder, USA; Auckland Bioengineering Institute, University of Auckland, Auckland, New Zealand; Royal College of Arts, London, UK; Medical Informatics Laboratory, University Medicine Greifswald, Greifswald, Germany; Department of Computer and Information Science, University of Konstanz, Konstanz, Germany; Faculty of Information Technology, Monash University, Clayton, Australia

## Abstract

This special issue of the Journal of Integrative Bioinformatics contains updated specifications of COMBINE standards in systems and synthetic biology. The 2022 special issue presents three updates to the standards: CellML 2.0.1, SBML Level 3 Package: Spatial Processes, Version 1, Release 1, and Synthetic Biology Open Language (SBOL) Version 3.1.0. This document can also be used to identify the latest specifications for all COMBINE standards. In addition, this editorial provides a brief overview of the COMBINE 2022 meeting in Berlin.

**Research funding:** This work was funded by German Research Foundation (DFG) 436883643, (DFG) 465194077, BMBF 031L0304B and EOSC 101017536.

***Corresponding author: Matthias König**, Institute for Biology, Institute for Theoretical Biology, Humboldt-University Berlin, Berlin, Germany, E-mail: koenigmx@hu-berlin.de. https://orcid.org/0000-0003-1725-179X
**Padraig Gleeson and Sarah M. Keating**, University College London, London, UK. https://orcid.org/0000-0001-5963-8576 (P. Gleeson), https://orcid.org/0000-0002-3356-3542 (S. M. Keating)
**Martin Golebiewski**, Heidelberg Institute for Theoretical Studies (HITS), Heidelberg, Germany. https://orcid.org/0000-0002-8683-7084
**Thomas E. Gorochowski**, School of Biological Sciences, University of Bristol, Bristol, UK. https://orcid.org/0000-0003-1702-786X
**Michael Hucka**, California Institute of Technology, Pasadena, USA. https://orcid.org/0000-0001-9105-5960
**Chris J. Myers**, Department of Electrical, Computer, and Energy Engineering, University of Colorado Boulder, Boulder, USA
**David P. Nickerson**, Auckland Bioengineering Institute, University of Auckland, Auckland, New Zealand. https://orcid.org/0000-0003-4667-9779
**Björn Sommer**, Royal College of Arts, London, UK
**Dagmar Waltemath**, Medical Informatics Laboratory, University Medicine Greifswald, Greifswald, Germany. https://orcid.org/0000-0002-5886-5563
**Falk Schreiber**, Department of Computer and Information Science, University of Konstanz, Konstanz, Germany; and Faculty of Information Technology, Monash University, Clayton, Australia

## Specifications of Standards in Systems and Synthetic Biology: Status and Developments in 2022 and the COMBINE meeting 2022

**Matthias König[1*], Padraig Gleeson[2], Martin Golebiewski[3], Thomas E. Gorochowski[4], Michael Hucka[5], Sarah M. Keating[2], Chris J. Myers[6], David P. Nickerson[7], Björn Sommer[8], Dagmar Waltemath[9], and Falk Schreiber[10,11]**

[1]Institute for Biology, Institute for Theoretical Biology, Humboldt-University Berlin, Germany

[2]University College London, UK

[3]Heidelberg Institute for Theoretical Studies (HITS), Germany

[4]School of Biological Sciences, University of Bristol, UK

[5]California Institute of Technology, USA

[6]Dept. of Electrical, Computer, and Energy Eng., University of Colorado Boulder, USA

[7]Auckland Bioengineering Institute, University of Auckland, New Zealand

[8]Royal College of Arts, London, UK

[9]University Medicine Greifswald, Germany

[10]Dept. of Computer and Information Science, University of Konstanz, Germany

[11]Faculty of Information Technology, Monash University, Clayton, Australia

[*]To whom correspondence should be addressed. Email:koenigmx@hu-berlin.de

### Abstract

This special issue of the Journal of Integrative Bioinformatics contains updated specifications of COMBINE standards in systems and synthetic biology. The 2022 special issue presents three updates to the standards: CellML 2.0.1, SBML Level 3 Package: Spatial Processes, Version 1, Release 1, and Synthetic Biology Open Language (SBOL) Version 3.1.0. This document can also be used to identify the latest specifications for all COMBINE standards. In addition, this editorial provides a brief overview of the COMBINE 2022 meeting in Berlin.

### 1 Introduction

The "COmputational Modeling in BIology NEtwork" (COMBINE) is an initiative to coordinate the development of the various community standards and formats for computational models [1]. By doing so, it is expected that the federated projects will develop a set of interoperable and non-overlapping standards covering all aspects of modeling in biology.

Figure 1 shows an overview of the COMBINE standards and associated initiatives. This editorial presents the latest specifications of all COMBINE standards, and this special issue highlights the updates of the last year, namely the releases of the following specifications: CellML 2.0.1, SBML Level 3 Package: Spatial Processes, Version 1, Release 1, and Synthetic Biology Open Language (SBOL) Version 3.1.0. Special issues on COMBINE standards have been published since 2016, and earlier editions [3, 4, 2, 5, 6, 7] provide updates for the years 2015 to 2021. In addition, this editorial provides a brief overview of the COMBINE 2022 meeting.

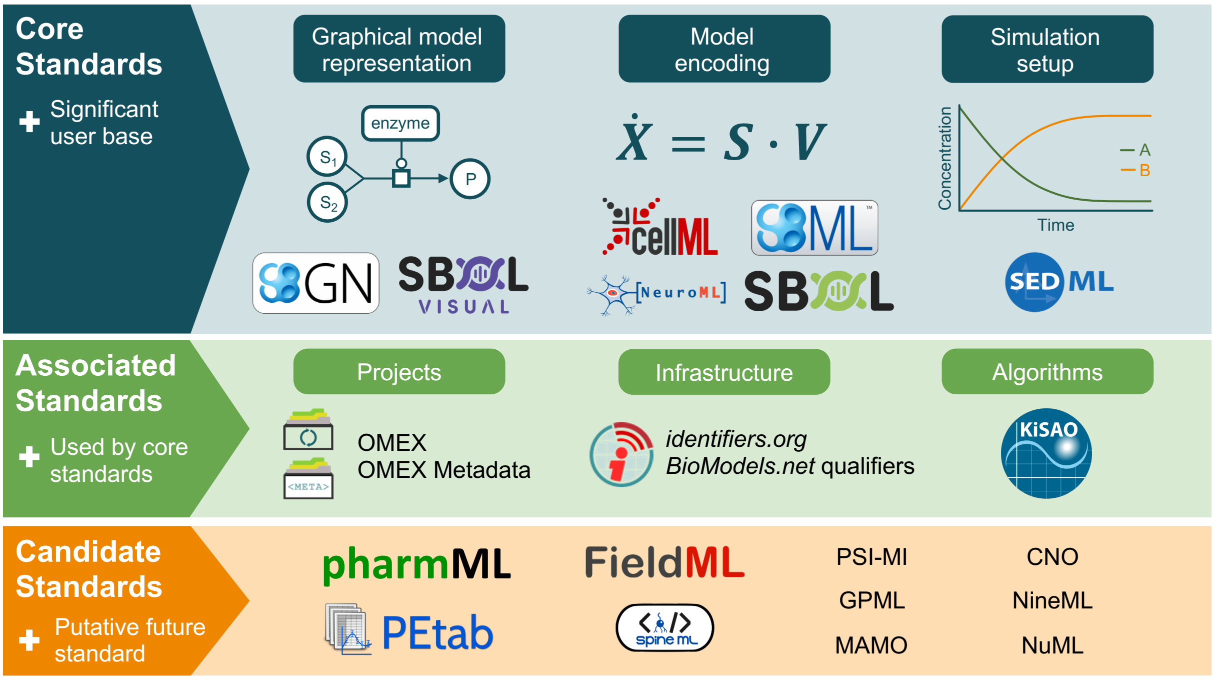

**Figure 1: COMBINE standards and associated efforts (updated from [2]).**

### 2 COMBINE meeting 2022 Berlin

COMBINE 2022 was a three-day workshop-style event held October 6-8, 2022, at the Institute of Biology/ITB of the Humboldt University of Berlin (see Figure 2). The meeting was organized by Matthias König and Dagmar Waltemath as a satellite event to the ICSB 2022 meeting in Berlin. The program, list of abstracts and participants are available from https://github.com/matthiaskoenig/combine2022.

The main focus of COMBINE 2022 was to coordinate the development of community standards and formats in systems biology and related fields. The meeting featured oral presentations, breakout sessions, and tutorials covering a wide range of topics related to the COMBINE standards, associated or related standardisation efforts, and tools using these standards.

Some of the topics covered during the meeting included the latest developments in

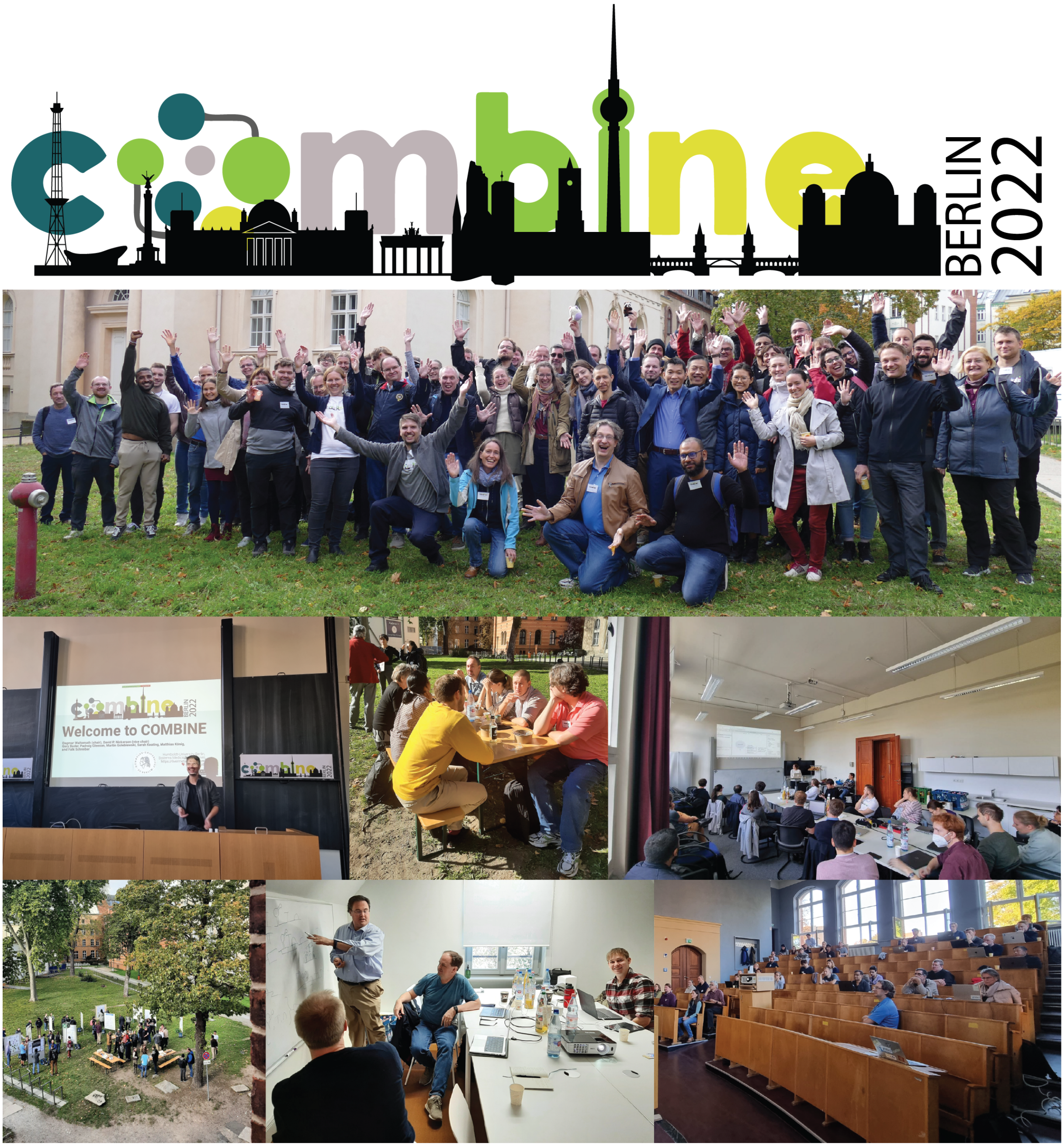

**Figure 2: COMBINE meeting 2022 at the Humboldt-University Berlin.**

the Systems Biology Markup Language (SBML), CellML, NeuroML, and the Simulation Experiment Description Markup Language (SED-ML). Other topics included the use of FAIR (Findable, Accessible, Interoperable, and Reusable) principles in computational modeling and the use of ontologies in systems biology.

The meeting provided attendees with the opportunity to learn from experts and enthusiasts in systems biology and related fields. The breakout sessions allowed for in-depth discussions of specific topics, while the tutorials provided hands-on training in the use of the COMBINE standards and related tools.

Overall, COMBINE 2022 was a successful event with more than 100 attendees from around the world, bringing together researchers, developers, and users of the COMBINE standards to collaborate and advance the field of systems biology and related areas. We would like to thank all participants of COMBINE 2022 for making it an engaging and highly productive event.

### 3 Current versions of COMBINE standards

As in the previous special issues in the following we will give a brief overview of all COMBINE standards. When using COMBINE standards, please refer to the following (most up-to-date) specifications. New specifications or updates of existing specifications are highlighted with **NEW**.

### 3.1 Core Standards

#### 3.1.1 BioPAX (Biological PAthway eXchange)

BioPAX is a standard language for integration, exchange and analysis of biological pathway data. It is expressed in OWL. The current specification is:

| Standard | Specification | Reference |
| --- | --- | --- |
| BioPAX [8] | BioPAX | [9] |

#### 3.1.2 CellML

The CellML language is an XML markup language to store and exchange computer-based mathematical models. The current specifications are listed in the following table.

**NEW** CellML 2.0.1 [10] updates CellML 2.0 [11] with an improved identification of rule statements and corrects errata present in the CellML 2.0 specification.

| Standard | Specification | Reference |
| --- | --- | --- |
| CellML [12] | CellML 2.0.1 | [10] |
| | CellML Metadata Framework 2.0 | [13] |
| | CellML 1.1 | [14] |

#### 3.1.3 NeuroML

The Neural Open Markup Language (NeuroML) is an XML-based description language that provides a common data format for defining and exchanging descriptions of neuronal cell and network models. The current specification is:

| Standard | Specification | Reference |
|---|---|---|
| NeuroML [15, 16] | NeuroML version 2.1 | [15] |

#### 3.1.4 SBGN (Systems Biology Graphical Notation)

The Systems Biology Graphical Notation (SBGN), is a set standard graphical languages to describe visually biological knowledge. It consists of three languages describing Process Descriptions, Entity Relationships and Activity Flows. In addition, SBGN-ML is a XML-based file format describing the geometry of SBGN maps, while preserving their underlying biological meaning. The current specifications are:

| Standard | Specification | Reference |
|---|---|---|
| SBGN [17] | SBGN Process Description Level 1 Version 2 | [18] |
| | SBGN Entity Relationship Level 1 Version 2.0 | [19] |
| | SBGN Activity Flow Level 1 Version 1.2 | [20] |
| | SBGN Markup Language Version 0.3 | [21] |

#### 3.1.5 SBML (Systems Biology Markup Language)

The Systems Biology Markup Language (SBML) is a computer-readable XML format for representing models of biological processes. SBML is suitable for, but not limited to, models using a process description approach. SBML development is coordinated by an elected editorial board and central developer team. The current specifications are listed in the following table.

**NEW** SBML Level 3 Package: Spatial Processes, Version 1, Release 1 [22] adds the necessary features to SBML to allow models to encode geometries and other spatial information about the elements and processes it describes.

| **Standard** | **Specification** | **Reference** |
|---|---|---|
| SBML [23] | SBML Level 3 Core, Version 2, Release 2 | [24] |
| | SBML Level 3 Package: Distributions, Version 1, Release 1 | [25] |
| | SBML Level 3 Package: Flux Balance Constraints Version 2, Release 1 | [26] |
| | SBML Level 3 Package: Groups, Version 1, Release 1 | [27] |
| | SBML Level 3 Package: Hierarchical Model Composition, Version 1, Release 3 | [28] |
| | SBML Level 3 Package: Layout, Version 1, Release 1 | [29] |
| | SBML Level 3 Package: Multistate, Multicomponent and Multicompartment Species, Version 1, Release 2 | [30] |
| | SBML Level 3 Package: Spatial Processes, Version 1, Release 1 | [22] |
| | SBML Level 3 Package: Qualitative Models, Version 1, Release 1 | [31] |
| | SBML Level 3 Package: Render, Version 1, Release 1, Release 1 | [32] |

#### 3.1.6 SBOL (Synthetic Biology Open Language)

The Synthetic Biology Open Language (SBOL) is a language for the description and the exchange of synthetic biological parts, devices, and systems. SBOL Visual (SBOLv) is a complementary standard that provides a standard set of glyphs and rules for drawing genetic circuit diagrams. The current specifications are listed in the following table.

**NEW** Synthetic Biology Open Language (SBOL) Version 3.1.0 [33] is minor revision of the SBOL standard, which improves on version 3.0.0 by including a number of corrections and clarifications as well as several other updates and enhancements.

| **Standard** | **Specification** | **Reference** |
|---|---|---|
| SBOL [34] | SBOL Version 3.1.0 | [33] |
| | SBOL Visual Version 2.3 | [35] |
| | SBOL Visual Version 3.0 | [36] |

#### 3.1.7 SED-ML (Simulation Experiment Description Markup Language)

The Simulation Experiment Description Markup Language is an XML-based format for encoding simulation experiments. SED-ML allows to define the models to use, the experimental tasks to run and which results to produce. SED-ML can be used with models encoded in several languages. The current specification is:

| **Standard** | **Specification** | **Reference** |
|---|---|---|
| SED-ML [37] | SED-ML Level 1 Version 4 | [38] |

### 3.2 Associated Standards

Associated standards provide an additional layer of semantics to COMBINE representation formats. The current specifications are:

| Standard | Specification | Reference |
|---|---|---|
| COMBINE Archive [39] | COMBINE Archive 1.0 | [40] |
| OMEX Metadata | OMEX Metadata Version 1.2 | [41] |
| BioModels.net qualifiers [42] | - | [43] |
| Identifiers.org URIs [44] | - | [45] |
| Systems Biology Ontology [46] | [external] Bioportal | [47] |
| Kinetic Simulation Algorithm Ontology [46] | [external] Bioportal | [48] |

A COMBINE archive is a single file bundling the various documents and all relevant information necessary for a modelling and simulation project. The archive is encoded using the Open Modeling EXchange format (OMEX).

COMBINE archive metadata provides a harmonised, community-driven approach for annotating a variety of standardised model and data representation formats within a COMBINE archive.

BioModels.net qualifiers are standardised relationships (predicates) that specify the relation between an object represented in a description language and the external resource used to annotate it. MIRIAM Unique Resource Identifiers allow one to uniquely and unambiguously identify an entity in a stable and perennial manner. MIRIAM Registry is a set of services and resources that provide support for generating, interpreting and resolving MIRIAM URIs. Through the Identifiers.org technology, MIRIAM URIs can be dereferenced in a flexible and robust way. MIRIAM URIs are used by SBML, SED-ML, CellML and BioPAX controlled annotation schemes.

The Systems Biology Ontology (SBO) is a set of controlled, relational vocabularies of terms commonly used in Systems Biology, and in particular in computational modelling. Each element of an SBML file carries an optional attribute sboTerm which value must be a term from SBO. Each symbol of SBGN is associated with an SBO term.

The Kinetic Simulation Algorithm Ontology (KiSAO) describes existing algorithms and their inter-relationships through their characteristics and parameters. KiSAO is used in SED-ML, which allows simulation software to automatically choose the best algorithm available to perform a simulation and unambiguously refer to it.

The OMEX Metadata Specification is a technical implementation of the community consensus across COMBINE standards to harmonise the way we describe computational models and other resources with metadata [49].

### Acknowledgements

## References

[1] D. Waltemath, M. Golebiewski, M. L. Blinov et al. The first 10 years of the international coordination network for standards in systems and synthetic biology (COMBINE). *Journal of Integrative Bioinformatics*, 17(2-3):20200005, 2020.

[2] F. Schreiber, G. D. Bader, P. Gleeson et al. Specifications of standards in systems and synthetic biology: Status and developments in 2018. *Journal of Integrative Bioinformatics*, 15(1):13, 2018.

[3] F. Schreiber, G. D. Bader, M. Golebiewski et al. Specifications of standards in systems and synthetic biology. *Journal of Integrative Bioinformatics*, 12(2):258, 2015.

[4] F. Schreiber, G. D. Bader, P. Gleeson, M. Golebiewski, M. Hucka, N. L. Novère, C. Myers, D. Nickerson, B. Sommer and D. Waltemath. Specifications of standards in systems and synthetic biology: Status and developments in 2016. *Journal of Integrative Bioinformatics*, 13(3):289, 2016.

[5] F. Schreiber, B. Sommer, G. D. Bader et al. Specifications of standards in systems and synthetic biology: Status and developments in 2019. *Journal of Integrative Bioinformatics*, 16(2):35, 2019.

[6] F. Schreiber, B. Sommer, T. Czauderna et al. Specifications of standards in systems and synthetic biology: status and developments in 2020. *Journal of Integrative Bioinformatics*, 17(2-3), 2020.

[7] F. Schreiber, P. Gleeson, M. Golebiewski et al. Specifications of standards in systems and synthetic biology: Status and developments in 2021. *Journal of Integrative Bioinformatics*, 18(3):20210026, 2021.

[8] E. Demir, M. P. Cary, S. Paley et al. The BioPAX community standard for pathway data sharing. *Nature Biotechnology*, 28:935–942, 2010.

[9] BioPax, 2017. http://www.biopax.org/.

[10] M. Clerx, M. T. Cooling, J. Cooper, A. Garny, K. Moyle, D. P. Nickerson, P. M. F. Nielsen and H. Sorby. CellML 2.0.1. *Journal of Integrative Bioinformatics*, JIB.2023.0003, 2023.

[11] M. Clerx, M. T. Cooling, J. Cooper, A. Garny, K. Moyle, D. P. Nickerson, P. M. F. Nielsen and H. Sorby. CellML 2.0. *Journal of Integrative Bioinformatics*, 17(2):21, 2020.

[12] A. A. Cuellar, C. M. Lloyd, P. F. Nielsen, D. Bullivant, D. Nickerson and P. Hunter. An overview of CellML 1.1, a biological model description language. *Simulation*, 79(12):740–747, 2003.

[13] M. T. Cooling and P. J. Hunter. The CellML metadata framework 2.0 specification. *Journal of Integrative Bioinformatics*, 12(2):260, 2015.

[14] A. A. Cuellar, W. Hedley, M. Nelson, C. M. Lloyd, M. D. B. Halstead, D. P. Bullivant, D. P. Nickerson, P. J. Hunter and P. M. F. Nielsen. The CellML 1.1 specification. *Journal of Integrative Bioinformatics*, 12(2):259, 2015.

[15] R. C. Cannon, P. Gleeson, S. Crook, G. Ganapathy, B. Marin, E. Piasini and R. A. Silver. LEMS: A language for expressing complex biological models in concise and hierarchical form and its use in underpinning NeuroML 2. *Frontiers in Neuroinformatics*, 8(79), 2014.

[16] P. Gleeson, S. Crook, R. C. Cannon et al. NeuroML: A language for describing data driven models of neurons and networks with a high degree of biological detail. *PLoS Computational Biology*, 6(6):e1000815, 2010.

[17] N. Le Novère, M. Hucka, H. Mi et al. The Systems Biology Graphical Notation. *Nature Biotechnology*, 27(8):735–741, 2009.

[18] A. Rougny, V. Toure, S. Moodie et al. Systems biology graphical notation: Process description language level 1 version 2. *Journal of Integrative Bioinformatics*, 16(3):22, 2019.

[19] A. A. Sorokin, N. Le Novère, A. Luna, T. Czauderna, E. Demir, R. Haw, H. Mi, S. L. Moodie, F. Schreiber and A. Villéger. Systems biology graphical notation: Entity relationship language level 1 version 2. *Journal of Integrative Bioinformatics*, 12(2):264, 2015.

[20] H. Mi, F. Schreiber, S. L. Moodie, T. Czauderna, E. Demir, R. Haw, A. Luna, N. Le Novère, A. A. Sorokin and A. Villéger. Systems biology graphical notation: Activity flow language level 1 version 1.2. *Journal of Integrative Bioinformatics*, 12(2):265, 2015.

[21] F. T. Bergmann, T. Czauderna, U. Dogrusoz, A. Rougny, A. Dräger, V. Toure, A. Mazein, M. L. Blinov and A. Luna. Systems Biology Graphical Notation Markup Language (SBGNML) Version 0.3. *Journal of Integrative Bioinformatics*, 17(2):16, 2020.

[22] J. Schaff, A. Lakshminarayana, R. Murphy, F. Bergmann, A. Funahashi, D. Sullivan and L. P. Smith. SBML Level 3 Package: Spatial Processes, Version 1, Release 1. *Journal of Integrative Bioinformatics*, JIB.2022.0054, 2023.

[23] M. Hucka, A. Finney, H. M. Sauro et al. The Systems Biology Markup Language (SBML): A medium for representation and exchange of biochemical network models. *Bioinformatics*, 19(4):524–531, 2003.

[24] M. Hucka, F. T. Bergmann, C. Chaouiya et al. The Systems Biology Markup Language (SBML): Language Specification for Level 3 Version 2 Core Release 2. *Journal of Integrative Bioinformatics*, 16(3):21, 2019.

[25] L. P. Smith, S. L. Moodie, F. T. Bergmann, C. Gillespie, S. M. Keating, M. König, C. J. Myers, M. J. Swat, D. J. Wilkinson and M. Hucka. SBML Level 3 Package: Distributions, Version 1, Release 1. *Journal of Integrative Bioinformatics*, 17(2):18, 2020.

[26] B. G. Olivier and F. T. Bergmann. SBML level 3 package: Flux balance constraints, version 2. *Journal of Integrative Bioinformatics*, 15(1):82, 2018.

[27] M. Hucka and L. P. Smith. The Systems Biology Markup Language (SBML) Level 3 Package: Groups, Version 1 Release 1. *Journal of Integrative Bioinformatics*, 13(3):290, 2016.

[28] L. P. Smith, M. Hucka, S. Hoops, A. Finney, M. Ginkel, C. J. Myers, I. I. Moraru and W. Liebermeister. SBML level 3 package: Hierarchical model composition, version 1 release 3. *Journal of Integrative Bioinformatics*, 12(2):268, 2015.

[29] R. Gauges, U. Rost, S. Sahle, K. Wengler and F. T. Bergmann. The systems biology markup language (SBML) level 3 package: Layout, version 1 core. *Journal of Integrative Bioinformatics*, 12(2):267, 2015.

[30] F. Zhang, L. P. Smith, M. L. Blinov et al. SBML Level 3 package: Multistate, Multicomponent and Multicompartment Species, Version 1, Release 2. *Journal of Integrative Bioinformatics*, 17(2):15, 2020.

[31] C. Chaouiya, S. M. Keating, D. Bérenguier, A. Naldi, D. Thieffry, M. P. van Iersel, N. Le Novère and T. Helikar. The systems biology markup language (SBML) level 3 package: Qualitative models, version 1, release 1. *Journal of Integrative Bioinformatics*, 12(2):270, 2015.

[32] F. T. Bergmann, S. M. Keating, R. Gauges, S. Sahle and K. Wengler. SBML level 3 package: Render, version 1, release 1. *Journal of Integrative Bioinformatics*, 15(1):78, 2018.

[33] L. Buecherl, T. Mitchell, J. Scott-Brown et al. Synthetic Biology Open Language (SBOL) Version 3.1.0. *Journal of Integrative Bioinformatics*, JIB.2022.0058, 2023.

[34] M. Galdzicki, K. P. Clancy, E. Oberortner et al. The Synthetic Biology Open Language (SBOL) provides a community standard for communicating designs in synthetic biology. *Nature Biotechnology*, 32(6):545–550, 2014.

[35] H. Baig, P. Fontanarossa, V. Kulkarni et al. Synthetic Biology Open Language Visual (SBOL Visual) Version 2.3. *Journal of Integrative Bioinformatics*, 18(3):2020.0045, 2021.

[36] J. Beal, H. Baig, P. Fontanarrosa, J. A. McLaughlin, J. Scott-Brown, P. Vaidyanathan, T. Gorochowski, G. Misirli and C. Myers. Synthetic Biology Open Language Visual (SBOL Visual) Version 3.0. *Journal of Integrative Bioinformatics*, 18(3):2021.0013, 2021.

[37] D. Waltemath, R. Adams, F. T. Bergmann et al. Reproducible computational biology experiments with SED-ML – the Simulation Experiment Description Markup Language. *BMC Systems Biology*, 5(1):198, 2011.

[38] L. Smith, F. Bergmann, A. Garny, T. Helikar, J. Karr, D. Nickerson, H. Sauro and M. König. Simulation experiment description markup language (SED-ML): Level 1 version 4. *Journal of Integrative Bioinformatics*, 18(3):2021.0021, 2021.

[39] F. T. Bergmann, R. Adams, S. Moodie et al. COMBINE archive and OMEX format: one file to share all information to reproduce a modeling project. *BMC Bioinformatics*, 15(1):369, 2014.

[40] F. T. Bergmann, N. Rodriguez and N. Le Novère. COMBINE archive specification version 1. *Journal of Integrative Bioinformatics*, 12(2):261, 2015.

[41] J. H. Gennari, M. König, G. Misirli, M. L. Neal and D. P. Nickerson. OMEX Metadata Specification Version 1.2. *Journal of Integrative Bioinformatics*, 18(3):2021.0020, 2021.

[42] C. Li, M. Courtot, N. Le Novère and C. Laibe. Biomodels. net web services, a free and integrated toolkit for computational modelling software. *Briefings in bioinformatics*, 11(3):270–277, 2010.

[43] BioModels.net Qualifiers, 2020. http://co.mbine.org/standards/qualifiers.

[44] N. Juty, N. Le Novère and C. Laibe. Identifiers. org and miriam registry: community resources to provide persistent identification. *Nucleic Acids Research*, 40(D1):D580–D586, 2012.

[45] Identifiers.org, 2020. http://identifiers.org/.

[46] M. Courtot, N. Juty, C. Knüpfer et al. Controlled vocabularies and semantics in systems biology. *Molecular Systems Biology*, 7(1):543, 2011.

[47] Systems Biology Ontology on BioPortal, 2019. http://bioportal.bioontology.org/ontologies/SBO.

[48] Kinetic Simulation Algorithm Ontology on BioPortal, 2019. http://bioportal.bioontology.org/ontologies/KISAO.

[49] M. L. Neal, M. König, D. Nickerson et al. Harmonizing semantic annotations for computational models in biology. *Briefings in Bioinformatics*, 20(2):540–550, 2019.
